# Skeletal muscle and adipose tissue changes in the first phase of treatment of pediatric solid tumors

**DOI:** 10.1002/cam4.3584

**Published:** 2020-11-03

**Authors:** Lenat Joffe, Wei Shen, Grace Shadid, Zhezhen Jin, Elena J. Ladas

**Affiliations:** ^1^ Department of Pediatrics Division of Pediatric Hematology/ Oncology/ Stem Cell Transplant Columbia University Irving Medical Center New York NY USA; ^2^ Department of Pediatric Gastroenterology, Hepatology and Nutrition Institute of Human Nutrition; and MR Research Center Columbia University Irving Medical Center New York NY USA; ^3^ Institute of Human Nutrition Columbia University Irving Medical Center New York NY USA; ^4^ Department of Biostatistics Mailman School of Public Health Columbia University New York NY USA

**Keywords:** body composition, childhood cancer, nutrition, nutritional status, pediatric cancer, solid tumors

## Abstract

Body composition is increasingly recognized as an important factor in cancer outcomes. Use of computed tomography (CT) in cancer care provides the opportunity to accurately quantify whole‐body lean and adipose tissues from images at the third lumbar spine. We sought to substantiate the use of routinely captured, single‐slice chest CT images at the thoracic level for evaluation of skeletal muscle, residual lean tissue, and adiposity among pediatric solid tumor patients. We performed a retrospective analysis among children who underwent treatment for a solid tumor at Columbia University Irving Medical Center. Skeletal muscle (SM), residual lean tissue (RLT), and adipose tissue cross‐sectional areas (cm^2^) were analyzed at diagnosis and at first follow‐up for disease evaluation (6–14 weeks). Imaging analysis was performed utilizing slice‐O‐matic image analysis software. Of the 57 patients identified, 39 had chest CT imaging that included intervertebral level T12‐L1, and 22 also had concurrent imaging at L3. Correlation coefficients between body composition variables at T12‐L1 and L3 were strong (*r* = 0.93–0.98). Paired t‐test showed a significant decrease in SM (−4.2 ± 8.12, *p* = 0.003) and RLT (−10.7 ± 28.5, *p* = 0.025) as well as a trend toward a significant increase in visceral adipose tissue (3.10 ± 9.65, *p* = 0.052). Univariable analysis demonstrated a significant association between increasing age and increased SM loss (*β* = −0.496 with *SE* = 0.194, *p* = 0.011), and a lack of association between body mass index and body composition changes. We provide the first line of evidence that single‐slice images from routinely obtained chest CT scans provide a simple, readily available mechanism for assessing body composition in pediatric solid tumor patients. Adverse body composition changes were observed, particularly among adolescents and young adults.

Precis: Changes in body composition can be detected via routine CT images in pediatric patients undergoing treatment for solid tumors.

## Graphical abstract

Body composition is a growing area of interest. Several studies in adults with cancer have found that suboptimal body composition (increased fat, decreased muscle) is strongly associated with the efficacy of treatment, quality of life, and death. However, its use in clinical practice, especially pediatrics, is limited due to the lack of a readily available, sensitive indicator. We sought to substantiate the use of routinely collected CT images to measure body composition. Our results substantiate the use of CT images for the evaluation of body composition thereby creating new clinical and research opportunities in pediatric cancer care.

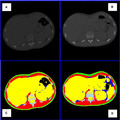

## 1 INTRODUCTION

There is a developing body of evidence indicating that nutritional status may impact treatment outcomes, including tolerance to chemotherapy, infection rates, prognosis, and quality of life, among pediatric solid tumor patients.^1^ In recent years, body composition, as a result of nutritional balance in conjunction with effects of the underlying disease, has been increasingly recognized as an important factor in both adult and pediatric cancer outcomes.^2^ Sarcopenia and sarcopenic obesity have consistently demonstrated an adverse relationship with treatment‐related complications, morbidity, and survival in the adult solid tumor population.^3^, ^4^, ^5^, ^6^, ^7^, ^8^, ^9^, ^10^ Body composition studies in pediatrics are limited, and current literature largely focuses on hematologic malignancies. Among those with acute lymphoblastic leukemia (ALL), dual‐energy X‐ray absorptiometry (DXA) has been the primary imaging modality employed for body composition analysis. Studies consistently demonstrate that ALL patients experience a significant decrease in skeletal muscle mass,^11^, ^12^ with a concomitant increase in fat mass^13^, ^14^ following the initiation of therapy. Similarly, pediatric studies utilizing nonimaging modalities to evaluate body composition in diverse groups of patients, including those with intracranial and extracranial solid tumors, show that this population as a whole experiences a decrease in fat‐free mass with concurrent increase in fat mass.^15^, ^16^, ^17^

Anthropometric measures such as body mass index (BMI), skin fold thickness, and mid‐upper arm or waist‐circumference, are easily obtained and often relied upon in the clinical setting.^2^ However, the utility of these modalities is limited due to their variability.^13^ Moreover, BMI does not distinguish between lean mass and adiposity,^3^, ^15^ and may even be inaccurate in patients with significant tumor burden; a clinical picture more likely in solid tumors. Use of advanced imaging techniques like computed tomography (CT) and magnetic resonance imaging (MRI) has enabled practitioners to accurately discern and quantify lean and adipose tissue compartments, and to further investigate their role in cancer outcomes.^2^ The utility of single‐slice CT images for body composition analysis, particularly at the vertebral level of L3, has been validated as an indicator of whole‐body tissue measurements in adult oncology literature.^7^, ^18^ Such methodology is advantageous in that it provides a readily available and practical means of body composition assessment, while circumventing the need for excess testing and/or exposure.^19^ Studies directed at supporting the use of single‐slice images for body tissue composition assessment are necessary to establish feasibility and utility of this technique in pediatric oncology.^20^ This study aims to substantiate the use of single‐slice images, captured via routinely performed on‐therapy chest CT scans, to assess body composition changes during the first 6 to 14 weeks of therapy among pediatric solid tumor patients.

## 2 METHODS AND MATERIALS

### 2.1 Study population

The records of children, adolescents, and young adults 1–21 years of age, who underwent treatment for a primary diagnosis of Wilms tumor, Ewing sarcoma, osteosarcoma, or rhabdomyosarcoma at Columbia University Irving Medical Center (CUIMC) between 2002 and 2017 were reviewed. Eligible participants were identified via applicable ICD‐9 and ICD‐10 diagnosis codes from the CUIMC institutional electronic medical records system. Patients of all risk stratification types and histologic subtypes were included in the study. Excluded were those who received radiotherapy during the study observation period, patients with relapsed or secondary malignancy, those enrolled in a phase I clinical trial, and any patients who did not have chest CT imaging available for both study time points (diagnosis and first follow‐up). All study participants were treated on or as per a Children's Oncology Group protocol during the study observation period (Table S1). In addition to body composition and nutritional anthropometrics (height, weight, BMI), demographic and treatment information including age, sex, self‐reported race and ethnicity, tumor grade and histology, treatment regimen, and survival data were retrospectively abstracted from medical records system. This study was approved by the CUIMC Institutional Review Board.

### 2.2 Assessment of body composition

**FIGURE 1:**
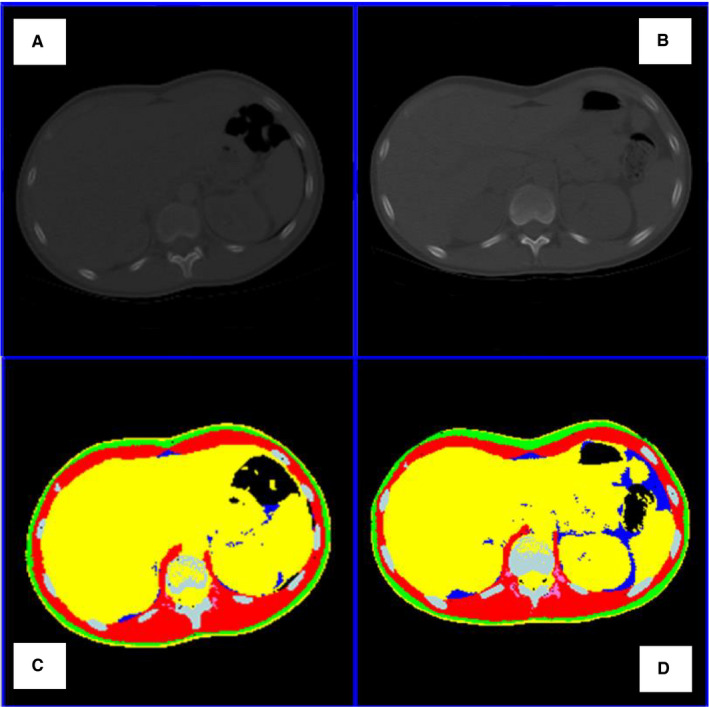
Single Slice Tissue Mass Quantification and Changes Over Time at the Level of T12‐L1 in an Adolescent Osteosarcoma Patient. A & B: Original chest CT scans at diagnosis (A) and following 2 cycles of neoadjuvant chemotherapy (B). C & D: Same images segmented, and each tissue tagged with a distinct color, allowing for tissue area quantification and comparison over time.

Body composition was assessed by the analysis of previously collected, electronically stored chest CT images obtained at two time points: diagnosis (prior to initiation of treatment with any antineoplastic agents) and first follow‐up disease evaluation (6 to 14 weeks after initiation of therapy, depending on disease treatment protocol, irrespective of upfront surgical resection). Measurement of tissue components including skeletal muscle (SM), visceral adipose tissue (VAT), subcutaneous adipose tissue (SAT), intermuscular adipose tissue (IMAT), and residual lean tissue (RLT) was performed on single‐slice images at a select anatomic landmark located in the intervertebral space between the 12th thoracic and first lumbar vertebras (T12‐L1). This anatomic location is typically captured as part of the chest CT, thereby eliminating the need for imaging beyond what is obtained in routine disease evaluation. Similar to the vertebral levels of L1 and L3 previously validated in adult cancer literature,^7^, ^21^ T12‐L1 captures the psoas, paraspinal, and abdominal wall muscles; as well as abdominal organs and fat.^18^ In patients whose scans captured L3, the same body components were analyzed at this level, and these measures were compared with the body component measurements at the T12‐L1 level (Figure 1).

All image analysis was performed at the Image Analysis Laboratory at CUIMC utilizing slice‐O‐matic image analysis software, version 5.0 (Tomovision, Montreal, Quebec, Canada). CT images were reviewed according to a standardized preanalysis quality control protocol. Incomplete images and those with severe artifacts, including out of field of view, were excluded. SM, VAT, SAT, and IMAT were identified and quantified by the use of Hounsfield unit (HU) thresholds (SM: –29 to +150; VAT, SAT, and IMAT: −190 to −30),^7^, ^22^ and all body tissue componentswere reported in cross‐sectional areas (cm^2^).

### 2.3 Nutritional data

Height, weight, and BMI were collected alongside body composition data, at the same designated study time points (diagnosis and first follow‐up). Anthropometric data collected within 14 days of the corresponding imaging was abstracted. For all patients, BMI percentiles were calculated as per the guidelines set forth by the Centers for Disease Control and Prevention and World Health Organization.^23^, ^24^ Patients were classified into four weight categories consisting of underweight (BMI ≤5th percentile), normal weight (BMI 6th–84th percentile), overweight (BMI 85‐94th percentile), and obese (BMI ≥95th percentile).

### 2.4 Statistical analysis

Categorical variables were presented as frequencies and percentages and continuous variables were expressed as mean ±*SD*. A paired *t*‐test was used for analysis of change in body composition measures between the two study time points. Pearson correlation coefficient was used to assess the correlation between measures at T12‐L1 and those at L3. Additionally, a generalized estimating equation (GEE) approach with linear link and working independence correlation was used to assess for factors associated with changes in body composition measures during follow‐up. Multivariable analysis was carried out with the factors *p* < 0.05 level in the univariable analyses. Kaplan–Meier analysis and logrank test were used to evaluate time to relapse, event‐free survival and deaths. A *p*‐value <0.05 was considered statistically significant. Statistical analyses were performed using SAS 9.4 software (SAS Institute).

## 3 RESULTS

### 3.1 Baseline characteristics of cohort

**FIGURE 2:**
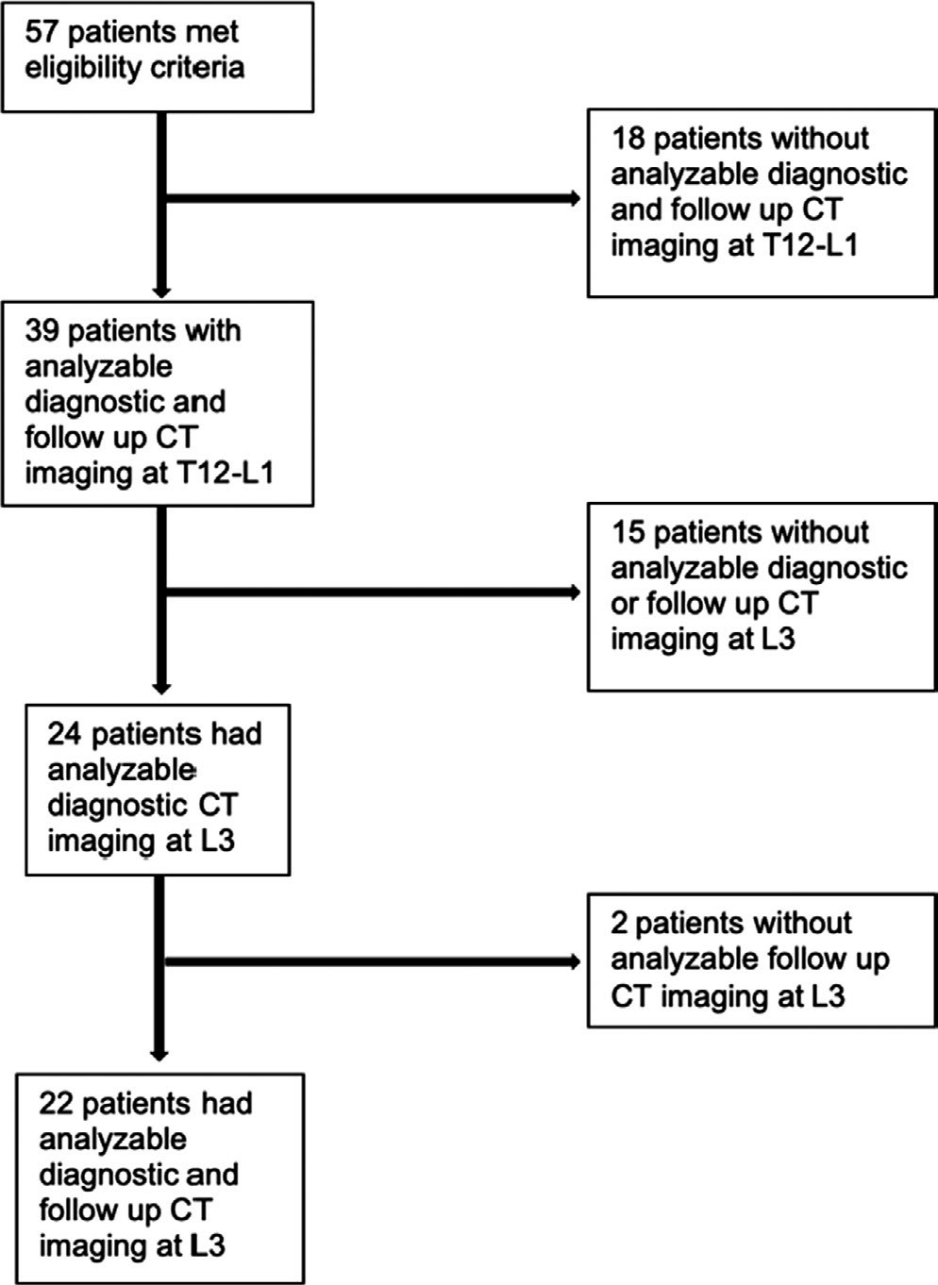
Study flow diagram

There were 57 patients who met initial inclusion criteria for our study. Of these, 39 patients (68.4%) had paired imaging that could be analyzed at T12‐L1 (mean timing between images 12.10 weeks, standard deviation 3.30), and 22 patients (38.6%) also had analyzable paired imaging at L3 for the same study time points (Figure 2). Mean age at diagnosis was 9.80 years (median, 11.0; range, 1.33–20.0 y). Participants were predominantly female (21/39), white (18/39), and non‐Hispanic (20/39). BMI data was available for 37 of the 39 patients at study entry as two patients’ records were archived in paper form and inaccessible for full review. Three patients (7.7%) were categorized as underweight and 15 patients (38.5%) were in the overweight or obese category (Table 1).

**TABLE 1:** Characteristics of study population

Characteristic	N (%)
Age (years)	
Mean, Median	9.80, 11.00
Minimum‐maximum	1.33–20.0
Sex	
Male	18 (46.2)
Female	21 (53.8)
Race	
White	18 (46.2)
Black	9 (23.1)
Asian/Pacific Islander	2 (5.1)
Other/Unknown	10 (25.6)
Ethnicity	
Hispanic	12 (30.8)
Non‐Hispanic	20 (51.3)
Unknown	7 (17.9)
Tumor type	
Ewing sarcoma	8 (20.5)
Osteosarcoma	7 (18)
Rhabdomyosarcoma	16 (41)
Wilms tumor	8 (20.5)
Body mass index category	
Underweight	3 (7.7)
Normal	19 (48.7)
Overweight/Obese	15 (38.5)
Unknown	2 (5.1)

### 3.2 Correlation of body composition assessment at T12‐L1 and L3 levels

There were 24 patients who had CT scans that included images at both T12‐L1 and L3 levels at baseline. Among these 24 patients, 22 also had CT scans that included images at both T12‐L1 and L3 levels at follow‐up. The correlation coefficients of body components measured at T12‐L1 and L3 ranged from 0.93 to 0.98 (Baseline, SM *r* = 0.98, VAT, *r* = 0.96; SAT, *r* = 0.98, IMAT, *r* = 0.95; all *p* < 0.0001; follow‐up, SM, *r* = 0.98, SAT, *r* = 0.96, IMAT, *r* = 0.92; VAT *r* = 0.95, all *p* < 0.0001) suggesting strong correlations between body tissue components at both imaging levels.

### 3.3 Body composition changes during therapy

Among the 39 patients for whom chest CT imaging was evaluable at T12‐L1, a statistically significant decrease in SM (−4.2 ± 8.12 cm^2^, *p* = 0.003) and RLT (−10.7 ± 28.5 cm^2^, *p* = 0.025) was observed between the two study time points. Moreover, this group exhibited a concomitant trend toward significantly increased VAT (3.10 ± 9.65 cm^2^, *p* = 0.052) throughout this time. For the subpopulation who also had imaging at L3, loss of SM was also statistically significant (−5.30 ± 11.7 cm^2^, *p* = 0.045), though the same did not hold true for RLT and VAT (Table 2). For BMI percentile, we found a reduction in the proportion of patients overweight/obese with a concomitant increase in participants classified as normal (among the 15 overweight/obese at diagnosis, five were reclassified as normal in follow‐up). Due to the area ranges of body composition variables being wider than anticipated, the cohort was then stratified by age (<12 years and ≥12 years). Loss of SM retained significance in the older population (n = 17 at T12‐L1; n = 11 at L3) at both T12‐L1 (*p* = 0.012) and L3 (*p* = 0.045). Yet, none of the body composition parameters remained significant in the younger group.

**TABLE 2:** Body composition changes over time

Variable (N)	Mean cm^2^ (SD)	Minimum‐maximum cm^2^	*p*‐value
T12‐L1 (39)			
SM	−4.20 (8.12)	−29.63–15.37	0.003
Muscle Density	−0.68 (5.57)	−12.10–14.99	0.450
SAT	1.06 (11.79)	−23.76–36.89	0.578
VAT	3.10 (9.65)	−8.20–50.95	0.052
IMAT	0.28 (2.23)	−3.10–11.21	0.445
TAT	4.44 (20.36)	−29.57–76.17	0.182
RLT	−10.65 (28.54)	−66.72–47.33	0.025
L3 (22)			
SM	−5.30 (11.66)	−35.26–10.53	0.045
Muscle Density	1.35 (4.53)	−4.70–10.06	0.175
SAT	−5.55 (18.10)	−40.17–35.88	0.165
VAT	3.09 (10.09)	−18.04–29.82	0.166
IMAT	−0.09 (0.70)	−2.14–1.22	0.543
TAT	−2.56 (23.40)	−51.80–59.13	0.614
RLT	−10.28 (37.81)	−66.42–56.71	0.216

Abbreviations: IMAT, intermuscular adipose tissue; RLT, residual lean tissue; SAT, subcutaneous adipose tissue; SM, skeletal muscle; TAT, total adipose tissue; VAT, visceral adipose tissue.

Univariable GEE analysis was conducted to assess for factors associated with changes in body composition measures. The considered factors were age at diagnosis, gender, and BMI. We found that increasing age was significantly associated with increased loss of SM at T12‐L1 (*β* = −0.496 with *SE* = 0.194, *p* = 0.011) as well as L3 (*β* = −0.882 with *SE* = 0.370, *p* = 0.017). Additionally, the group of patients who were ≥12 years of age had significantly greater SM loss than those who were <12 years at L3 (*β* = −8.78 with *SE* = 4.48, *p* = 0.050). We also observed a trend toward significance at T12‐L1 (*β* = −5.02 with *SE* = 2.65, *p* = 0.058). By the end of the study period, the number of patients in the underweight category decreased to two (5.1%) while the number of patients who were overweight or obese decreased to 12 (30.8%). Despite these findings, univariable GEE analysis failed to show any association between SM or RLT loss and fluctuations in BMI percentile or gender.

Within our cohort there were nine (23.1%) relapses and five (12.8%) deaths, making a total of 11 patient events (28.2%) over a mean follow‐up time of 1680.54 days (minimum 31 days, maximum 5380 days). Survival analysis did not reveal a significant association between event‐free survival (EFS) and change in any of the body composition parameters during the first phase of therapy. However, rate of change for VAT (cm^2^/day) did demonstrate a trend toward significance with EFS in this population (*p* = 0.056), suggesting an association between increased VAT and reduced EFS.

## 4 DISCUSSION

The use of advanced imaging modalities to accurately and objectively assess body composition in cancer patients has revolutionized our understanding of how body tissue compartments change during the course of therapy. This study demonstrates that there is a simple, readily available mechanism for assessing body composition in the pediatric solid tumor population; thereby paving the way for new opportunities for research in this understudied area. Use of single‐slice T12‐L1 images from routinely obtained chest CT scans to definitively assess SM area and quality, adiposity, and its distribution, as well as RLT area throughout therapy will enable investigators to better define and understand the effects of sarcopenia and sarcopenic obesity in this population. Our findings show that pediatric patients with solid tumors experience a significant decrease in SM and RLT early into the course of their therapy, and are at risk for a concurrent increase in VAT. Moreover, adolescents and young adults (AYAs) are at even greater risk for these deleterious changes as compared to younger children. Thus, body composition assessment may be most critical in disease groups that more commonly impact the AYA population. Importantly, our study clearly demonstrates the limitations of BMI, as changes in body composition parameters were not associated with this anthropometric indicator.

In contrast to adult literature, altered body composition was not associated with survival outcomes in our cohort. This is likely due to the small sample size of our study population, and inability to adequately power the survival analysis. Yet, despite this limitation, the trend toward significance between rate of change in VAT and EFS is intriguing, and reinforces the fact that survival studies merit further investigation in a larger cohort. This is particularly important as adult survivors of childhood cancer have a known predisposition for becoming overweight or obese, with a consequent myriad of chronic health conditions and increased odds of mortality.^25^, ^26^, ^27^, ^28^ VAT is believed to be the most pathogenic fat depot, with multiple endocrine, metabolic, and immunological functions.^29^, ^30^ Its accumulation increases susceptibility to ischemic heart disease and arterial hypertension, as well as a variety of malignancies, such as pancreas, colon, and breast.^31^ Thus, timely and accurate quantification of visceral adiposity changes during therapy may mitigate both short‐ and long‐term outcomes in this population.

Body composition is widely believed to influence cancer outcomes via its role in chemotherapy pharmacokinetics. Disparities in lean and adipose tissues are thought to alter chemotherapy volume of distribution, metabolism, and clearance of hydrophilic and/or lipophilic drugs from systemic circulation.^4^, ^32^, ^33^, ^34^, ^35^ Among adult oncology patients, sarcopenia has repeatedly demonstrated an adverse relationship with tolerance to treatment and prevalence of dose‐limiting toxicities.^18^, ^36^ In pediatrics, existing studies have largely focused on the impact of obesity, and there remains a lack of consensus regarding the impact of adiposity on chemotherapy dosing.^37^ However, several studies suggest that, depending on the drug, excess fat may result in either inadequate dosing and reduced efficacy or impaired clearance and excess drug toxicity.^34^, ^38^ The poor association between BMI and body composition changes demonstrated in this investigation further supports the notion that body surface area is insufficient for dosing chemotherapy. Moreover, while this study did not examine treatment‐related toxicities, the suggested relationship between VAT and survival may be mediated by altered chemotherapy metabolism consequent to underlying changes in body composition. Thus, dosing by body composition variables may provide a more accurate means of reducing treatment‐related toxicities and improving survival.

This study is not without limitations. Body tissue composition was evaluated utilizing two‐dimensional contrast‐enhancing CT images, and may not reflect the entire tissue volume. Yet, the methodology employed is in line with what has been successfully implemented in numerous, large‐scale adult cancer studies, and represents an initial step in its application in the pediatric solid tumor population. Pediatric solid tumors are a relatively rare and the small sample size of our heterogeneous population may limit the generalizability of our findings. However, given that this is a previously underexamined area of pediatric oncology, this study serves as a key step in establishing a standard, reproducible methodology for body composition analysis in this population. Additionally, anthropometric indicators of nutritional status such as skin fold thickness, and mid‐upper arm circumference, were not available for collection and could not be examined alongside imaging data. This will be included in future prospective studies. Although our study was not powered to examine the effects of specific treatment regimens on body composition, toxicities, and outcomes, this study provides the necessary framework to establish the significance of body composition in solid tumor outcomes, and promote its investigation in large, prospective clinical trials.

In conclusion, the results of this study eliminate a major barrier in body composition analysis among pediatric solid tumor patients, and emphasize the importance of promoting further investigation in this patient population. Prospective clinical trials focusing on body composition and short‐ as well as long‐term outcomes are warranted to further understand the role of body composition, particularly among AYAs. Furthering our knowledge in this arena will ultimately allow practitioners to optimize supportive care interventions, reduce toxicities, and improve survival for this vulnerable population.

## CONFLICT OF INTEREST

None to disclose.

## AUTHOR CONTRIBUTION

All listed authors meet the ICMJE criteria. We attest that all the authors contributed significantly to the creation of this manuscript, each having fulfilled criteria as established by the ICMJE.

## Supporting information

Table S1

## Data Availability

The data that support the findings of this study are available from the corresponding author upon reasonable request.
