# Supplementary material for: Skeletal muscle and adipose tissue changes in the first phase of treatment of pediatric solid tumors
Source: Cancer Med. 2020 Nov 3;10(1):15–22. doi: 10.1002/cam4.3584 (PMC7826460; doi:10.1002/cam4.3584)
Supplement: Supplementary file 1 — Table S1 [file CAM4-10-15-s001.docx]

Supplemental table 1: Treatment protocol

| Protocol | N(%) |
| --- | --- |
| Ewing sarcoma |  |
| COG AEWS1031 | 2 (5.13) |
| COG AEWS1221 | 1 (2.56) |
| COG AEWS0031 | 5 (12.82) |
| Osteosarcoma |  |
| COG AOST0331 | 5 (12.82) |
| POG/CCG 9754 | 1 (2.56) |
| COG AOST0121 | 1 (2.56) |
| Rhabdomyosarcoma |  |
| COG ARST0531 | 3 (7.70) |
| COG ARST0331 | 6 (15.38) |
| COG ARST0431 | 1 (2.56) |
| COG ARST08P1 | 1 (2.56) |
| POG 9602 | 1 (2.56) |
| POG 9803 | 1 (2.56) |
| Mix | 3 (7.70) |
| Wilms tumor |  |
| COG AREN0534 | 2 (5.13) |
| COG AREN0533 | 1 (2.56) |
| NWTS 5 | 1 (2.56) |
| NWTS 4941 | 3 (7.70) |
| Mix | 1 (2.56) |

CCG, Children’s Cancer Group; COG, Children’s Oncology Group; NWTS, National Wilms’ Tumor Study Group; POG, Pediatric Oncology Group
